# Genome-wide measurement of spatial expression in patterning mutants of *Drosophila melanogaster*

**DOI:** 10.12688/f1000research.9720.1

**Published:** 2017-01-12

**Authors:** Peter A. Combs, Michael B. Eisen

**Affiliations:** 1 Graduate Program in Biophysics, University of California, Berkley, USA; 2 Department of Molecular and Cell Biology and Howard Hughes Medical Institute, University of California, Berkeley, USA

**Keywords:** Drosophila melanogaster, embryo, Zelda, Hunchback, Bicoid

## Abstract

Patterning in the *Drosophila melanogaster *embryo is affected by multiple maternal factors, but the effect of these factors on spatial gene expression has not been systematically analyzed. Here we characterize the effect of the maternal factors Zelda, Hunchback and Bicoid by cryosectioning wildtype and mutant blastoderm stage embryos and sequencing mRNA from each slice. The resulting atlas of spatial gene expression highlights the intersecting roles of these factors in regulating spatial patterns, and serves as a resource for researchers studying spatial patterning in the early embryo. We identify a large number of genes with both expected and unexpected patterning changes, and through integrated analysis of transcription factor binding data identify common themes in genes with complex dependence on these transcription factors.

## Introduction

In the early *Drosophila melanogaster* embryo, the spatially restricted activity of several maternally deposited factors establishes the main body axes of the animal by triggering cascades of patterned gene expression. Until recently, however, it was not practical to systematically characterize the effects of these factors on patterned expression, as the dominant method for studying spatial expression, *in situ* hybridization, does not scale well.

We previously introduced a method for the genome-wide measurement of spatial patterns of expression in the *Drosophila* embryo based on cryosectioning individual embryos along the anteroposterior axes and sequencing the mRNA from each slice. Here we extend this method to characterize embryos mutant for three maternal transcription factors (TFs): Bicoid, Zelda, and Hunchback.

All of these factors are crucial for proper patterning of the embryo. Bicoid is the primary, maternally provided anterior specifier that directly regulates many key gap and pair-rule genes ^1– 4^. More recently, the role of Zelda (also known as vfl) has been identified as an important factor in establishing cis-regulatory chromatin domains of patterned genes ^5– 12^. Finally, Hunchback is both maternally and zygotically expressed, and helps to specify the expression domains and levels of various gap and pair-rule genes in the thorax ^4, 13, 14^. Although broad-reaching examinations of the effects of mutating these genes on their targets has not previously been possible, mutating *bicoid* results in the anterior adopting an anterior-like fate ^15, 16^, mutating *zelda* leads to shifts of expression patterns in time and space ^12^, and Hunchback binding has been implicated in multiple *eve* stripes, among many other expression patterns. ^4, 17^; all of these mutations are lethal. Given the crucial roles of each of these factors in spatial patterning, we expected that perturbing their levels would lead to widespread direct and indirect effects on patterned genes.

## Results

### Genome-wide atlases of the blastoderm stage of multiple dosage mutants

**Figure 1.**
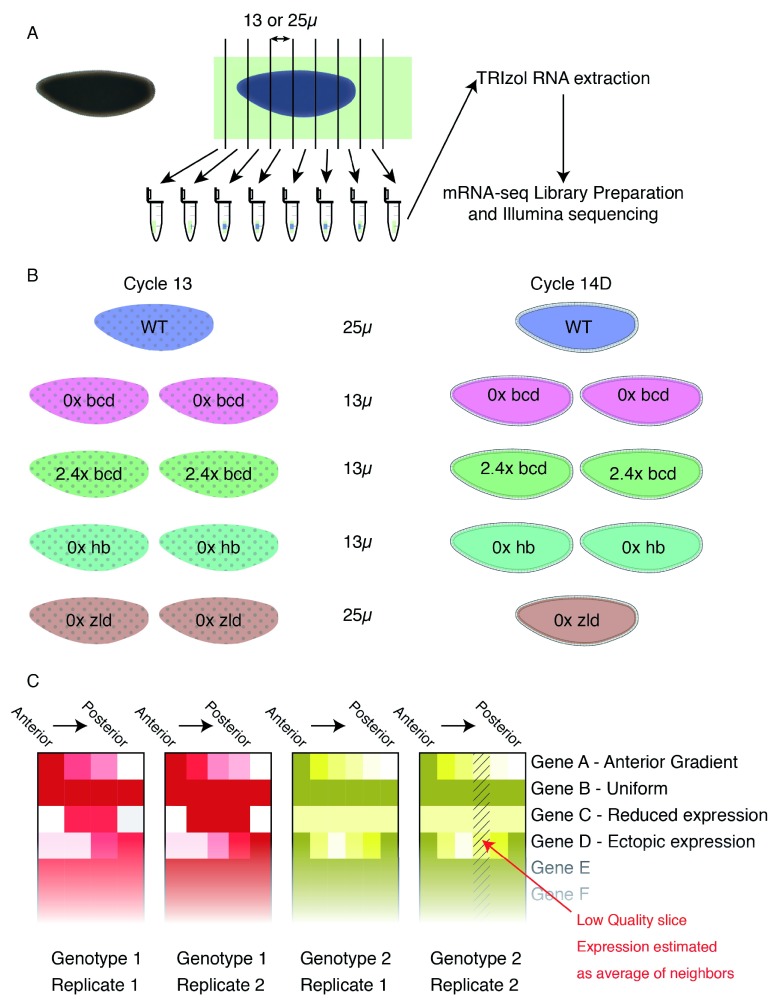
Schematic of experimental approach. **A**) We fixed embryos in methanol, then selected individual embryos at the correct stage, aligned them in sectioning cups, and sliced to the indicated thickness. We extracted RNA from individual slices, prepared barcoded libraries, then pooled them prior to sequencing. **B**) Overview of the mutant genotypes used. Two replicates per time point at two time points, based on nuclear density and morphology. **C**) Cartoon of heatmaps. Each genotype is assigned its own color (matching those in **B**), with darker colors representing higher expression and white representing no expression detected in that slice. Each boxed column represents a single individual, and within that column, slices are arranged posterior to the left and anterior to the right.

We sliced embryos and sequenced the resulting mRNA from 4 mutant genotypes ( Figure 1A): a *zld* germline clone, an RNAi knockdown for *bcd*, a knockdown for *hb*, and an overexpression line for *bcd* with approximately 2.4× wildtype expression. We chose two time points: cycle 13 (determined using nuclear density of either DAPI stained embryos or of the Histone-RFP present in the *zld* line) and mid-to-late cycle 14 (determined using 50–65% membrane invagination at stage 5) ( Figure 1B). Genes expressed in cycle 13 are towards the end of the early round of genome activation and are enriched for Zld binding ^10, 19, 19^, but are early enough that the majority of patterning disruptions are likely to be direct effects of the mutants. By contrast, we chose the stage 5 time point in order to highlight the full extent of the patterning changes across the network.

**Figure 2.**
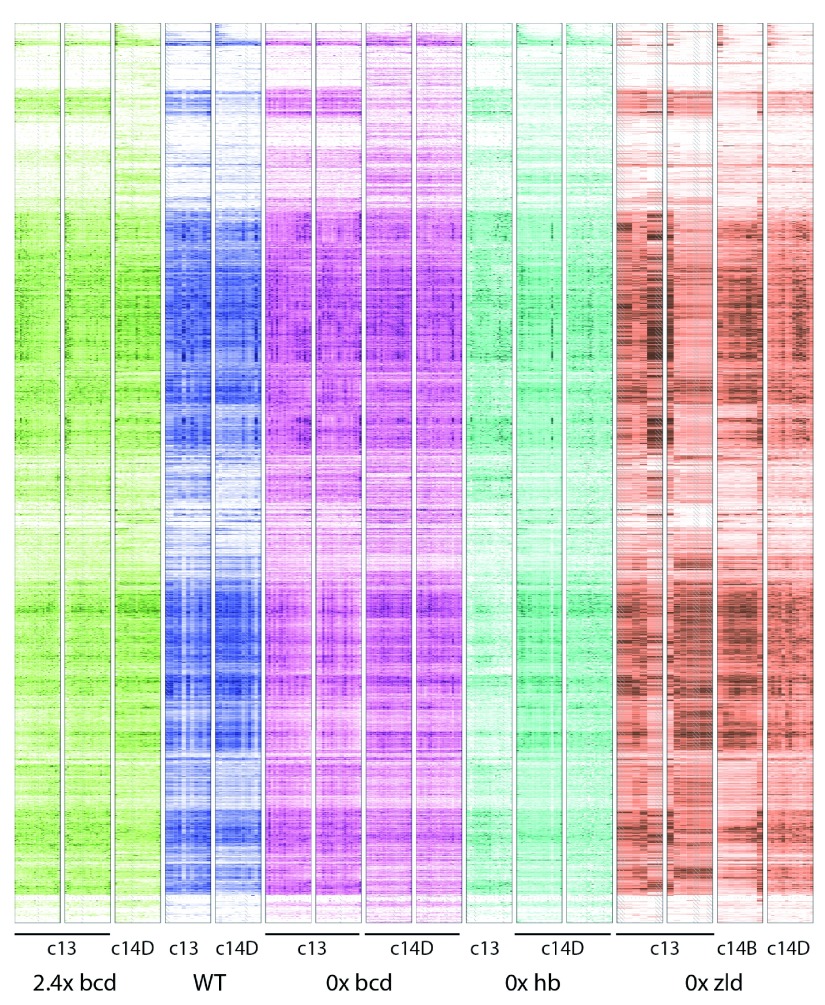
Heatmaps of gene expression patterns for all expressed genes. Each individual embryo is represented by one boxed column in the heatmap. Within a column, slices are arranged anterior to the left, and posterior to the right. Each embryo is colored according to genotype, with green for the *bcd* over-expression, blue for wild-type, magenta for *bcd* knockdown, cyan for *hb* knockdown, and orange for the *zld* mutant. Within a genotype, darker colors correspond to higher expression and white to zero expression, on a linear scale normalized for each gene separately to the highest expression for that gene in the embryo or to 10FPKM, whichever is greater. Slices that did not match quality control standards are replaced by averaging the adjacent slices, and are marked with hash marks. Rows are arranged by using Earth Mover’s Distance to perform hierarchical clustering, so that genes with similar patterns across all of the embryos are usually close together.

In order to show the range of patterning differences observed, we generated heatmaps of all the gene expression present in the dataset ( Figure 2). Of the 7104 genes with at least 15FPKM in at least one slice, approximately 3000 had uniform expression in all the wild-type embryos that was not greatly perturbed in any of the mutants. The total number of expressed genes is very consistent with previous estimates of the number of maternally deposited and zygotically transcribed genes ^19^.

**Figure 3.**
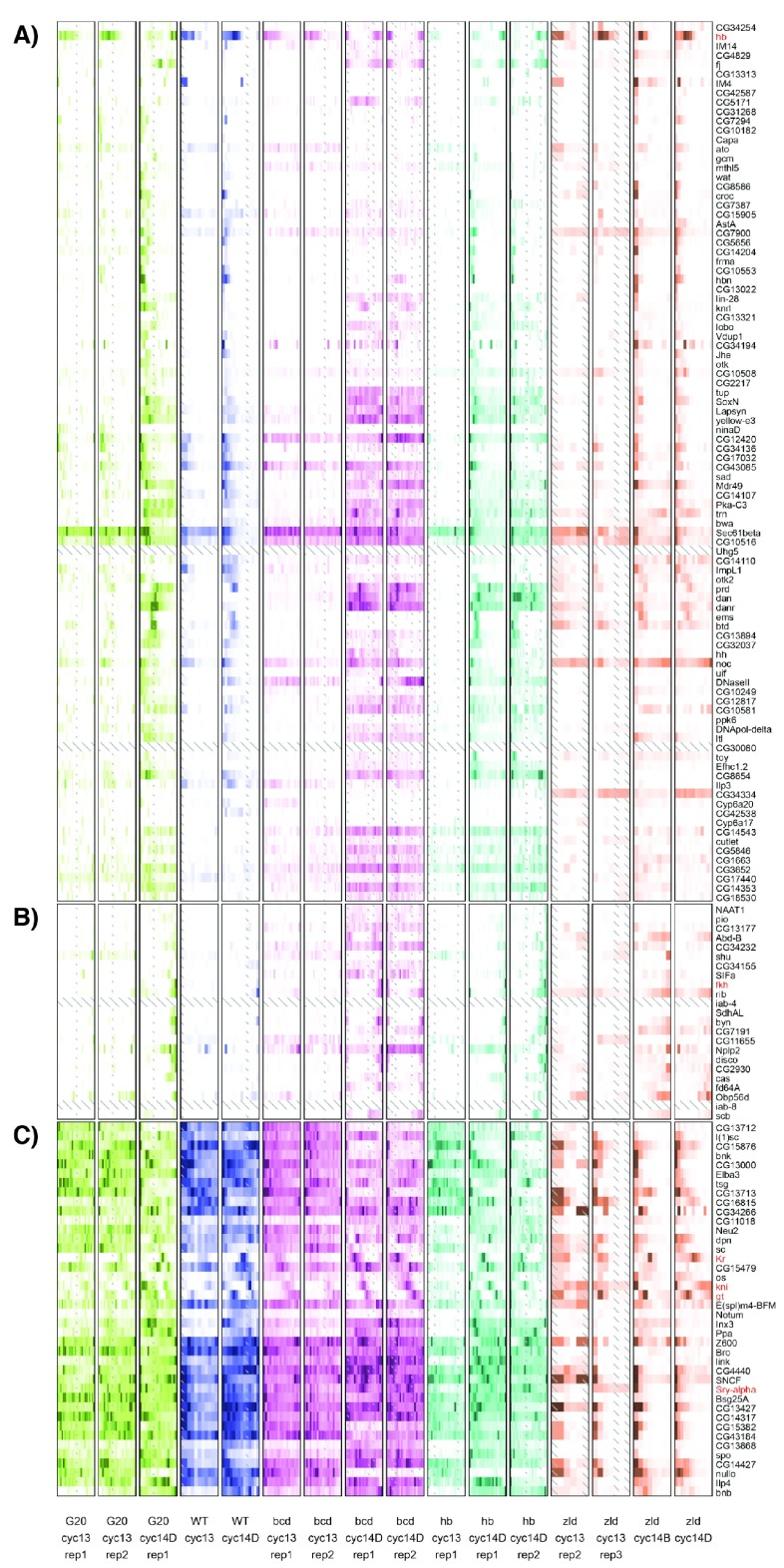
Heatmaps of gene expression patterns for anterior and posterior genes recapitulate expected patterning changes. We manually selected subsets of the larger heatmap in Figure 2 that showed clear differences between the genotypes. Each individual embryo is represented by one boxed column in the heatmap. Within a column, slices are arranged anterior to the left, and posterior to the right. Each embryo is colored according to genotype, with green for the *bcd* over-expression, blue for wild-type, magenta for *bcd* knockdown, cyan for *hb* knockdown, and orange for the *zld* mutant. Within a genotype, darker colors correspond to higher expression and white to zero expression, on a linear scale normalized for each gene separately to the highest expression for that gene in the embryo or to 10FPKM, whichever is greater. Genes identified in Liang *et al.* ^12^, Nien *et al.* ^53^, and Staller *et al.* ^16^ as responsive to either Bcd or Zld have red labels. **A**) Genes with anterior expression in the wild type embryos. **B**) Genes with posterior expression in wild type embryos. **C**) Genes with broad expression in wild type and anterior expression in later stage *zld-* embryos.

The set of genes with anterior or posterior localization recapitulate the known literature ^16^ and general expectations in the *bcd-* case: those expressed in the anterior typically lose expression ( Figure 3A), and those in the posterior also frequently gain an expression domain in the anterior ( Figure 3B). Surprisingly, most of these patterns are qualitatively unaffected in the other mutants. In the absence of *zld*, most of these genes are able to retain the proper anterior patterning (although they may have differences in expression levels). Similarly, these genes seem not to be strongly dependent on maternal Hb for patterning information, with most genes retaining a distinct anterior expression domain. As described in Liang *et al.* ^12^, there are some genes that are normally ubiquitously expressed in the wild-type that become localized to the poles in the *zld-* embryo ( Figure 3C).

### Most spatial patterns are robust to mutation

We next compared expression patterns from each of the mutant lines at late cycle 14 to similar expression patterns in wild-type. Because there are a different number of slices both between the wild type and mutant flies, and between replicates of the mutant flies, we decided to use Earth Mover’s Distance (EMD) to compare patterns ^20^. This metric captures intuitive notions about what kinds of patterns are dissimilar, yielding higher distances for dissimilar distributions of RNA, and zero for identical distributions. Patterns were normalized to have the same maximum expression, in order to highlight changes in positioning of patterns, rather than changes in absolute level. In contrast to traditional RNA-seq differential expression metrics, this approach takes advantage of the spatial nature of the data, and with the fine slices, adjacent slices are able to function as “pseudo-replicates”. Adjacent slices are, on average, much more similar than those from farther away in the same embryo ( Figure S1).

**Figure 4.**
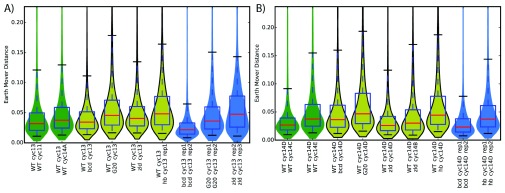
Distributions of patterning differences show that mutants have wide-spread subtle patterning effects and more genes with large patterning differences than replicates. Adjacent time points from the wild-type dataset in Combs and Eisen ^44^ are colored green, and replicates of the same genotype and time point are colored blue. Median distances are marked in red. **A**) Cycle 13 and **B**) mid cycle 14.

The overall level of divergence in pattern across all genes is, in most cases, slightly larger than when comparing nearby time-points in wild-type or replicates of the same genotype and time point ( Figure 4). Notably, the *zld-* mutant is more similar to wild-type than the mutants of the other, spatially distributed transcription factors. This suggests that Zld is a categorically different TF, consistent with its role as a pioneer factor rather than a direct activator. However, the low level of divergence is a reflection of the fact that the majority of genes are not dynamically expressed in either time or space.

**Figure 5.**
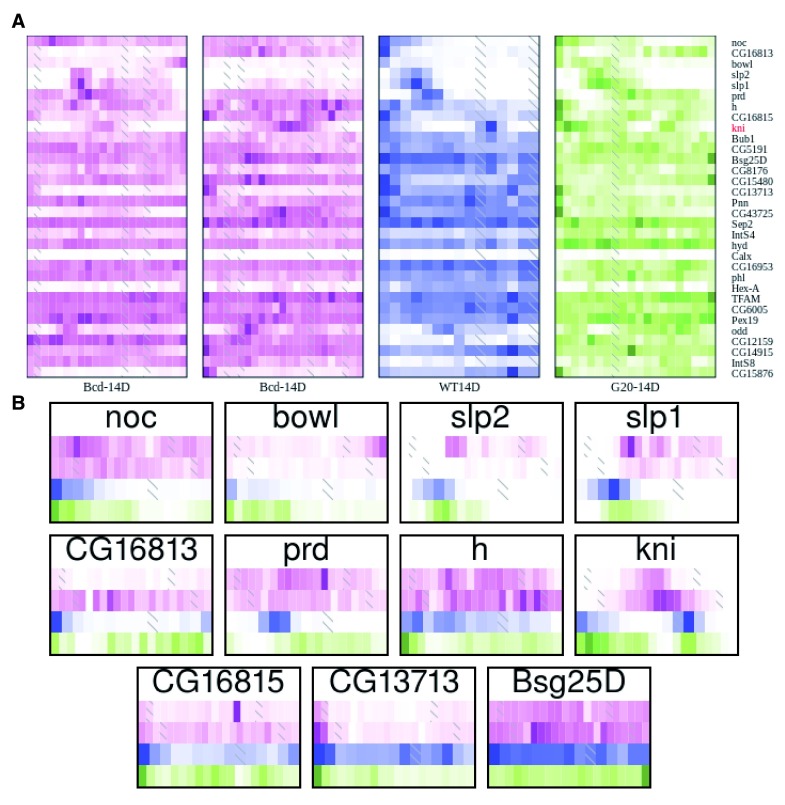
Patterning changes of genes near Bcd-dependent enhancers in *bcd* knockdown and overexpression are clearly visible in anterior-localized genes. **A**) Each individual is represented in its own heatmap. The magenta heatmaps are from the *bcd*- embryos, blue from wild-type, and green from 2.4× *bicoid.* **B**) Each gene with anterior localized expression in WT, with data from each individual as its own row, to highlight position changes across the mutant genotypes.

In order to demonstrate that these mutants are more likely to affect already known Bcd regulatory systems, we examined genes that were close to 64 Bcd dependent enhancers previously identified ^21– 27^. Although the bulk of these enhancers do not have validated associations with particular genes, we assumed that they would be relatively close to the genes that they drive. Of the 66 genes whose transcription start sites (TSSs) were the closest in either direction and within 10kb of the center of the tested CRM, only 32 were expressed at greater than 10FPKM in at least one slice of any of the wild-type embryos. Of these, only 10 had an obvious anterior localization bias (31%), with the majority of the rest being approximately uniformly expressed across the embryo ( Figure 5). The majority of genes with ubiquitious or central localization did not radically change in either the Bicoid overexpression or knockdown conditions. As expected, genes with anterior localization suffered a loss of patterning in the depletion mutant, and a posterior shift in the over-expression condition. We assume that genes that are not localized to the anterior are either driven by multiple enhancers, such that loss of expression from one does not severely affect the overall expression, or that they are merely close to the enhancer, but unrelated.

### Effects of TF depletion on patterned genes

**Figure 6.**
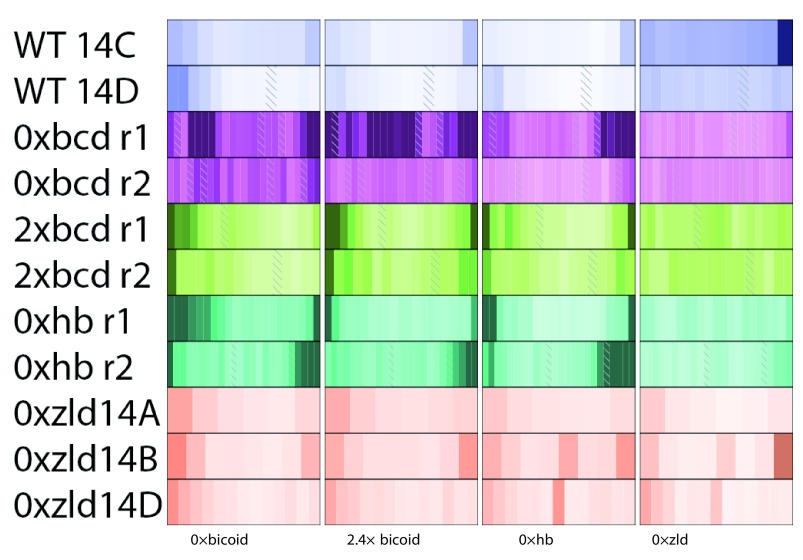
Averaging patterning changes in each genotype recapitulates known TF localization and function. Each individual is represented in its own heatmap. The magenta heatmaps are from the *bcd-* embryos, blue from wild-type, and green from 2.4× bicoid. A–D) The average pattern in each of the embryos of the 100 genes with the greatest change in *bcd* knockdown (A), *bicoid* overexpression (B), *hunchback* knockdown (C), and *zelda* knockout (D).

We next sought to demonstrate that the technique of cryoslicing mutants is useful for identifying the effects of these early patterning genes. In comparison to Figure 3, where we looked for known patterning changes that we would expect from the literature, we also want to make sure that the largest and most common patterning changes that naturally arise from the data recapitulate the known literature. For each mutant genotype, we identified the 100 genes with the largest patterning change in that genotype, then averaged the pattern in all of the cycle 14 embryos ( Figure 6).

Unsurprisingly, depletion of TFs known to be important for patterning are likely to make an otherwise non-uniform pattern more so ( Figure 1). Of the 465 genes that have clearly non-uniform patterning in the wild-type at cycle 14D, 12–20% are affected in each depletion mutant, either losing expression entirely or becoming uniform. The over-expression line is at the low end of this range, also at 12%.

**Table 1.** TF depletion is more likely to make a non-uniform pattern uniform than vice versa.

	Low Expr to Patterned	Patterned to Uniform	Patterned to Low Expr	Uniform to Patterned
2.4×bcd	343	32	25	64
0×bcd 0×hb 0 ×zld	96 348 71	69 36 34	40 20 61	12 43 28

We measured EMD for each gene at cycle 14D in each genotype compared to a uniform distribution. We considered genes uniform if they had an EMD<0.04, and non-uniform if they have an EMD>0.08. We then considered genes with at least 15 FPKM in at least one slice in both wild-type and the mutant line.

**Figure 7.**
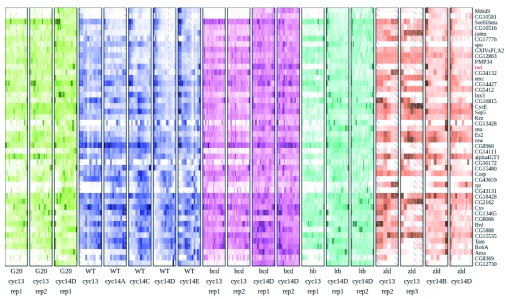
Patterned genes in wild-type that become uniformly expressed are widespread in *bcd-*. Each embryo is normalized independently.

However, this is not always simply abrogating expression—a large number of genes seem to have higher expression everywhere. In the case of *bcd* depletion, approximately a third of these cases are genes that are restricted to the anterior in wild-type that become approximately uniform throughout the embryo ( Figure 7). While some of these are due to genes with an early uniform pattern that fails to properly resolve into spatially restricted domains, approximately half are true ectopic expression ( Figure S2).

As a first step to identifying likely regulatory motifs, we used binding data for 9 non-pair-rule AP TFs ^28^ and for Zld ^10^ to search for factors with differential rates of binding among the sets of genes with patterning changes ( Figure 2). This analysis highlights that Zelda operates in a qualitatively different manner from the other transcription factors—in it’s absence other TFs are likely to continue expression, though in abnormal patterns. Additionally, Zld is crucial for maintaining patterned expression, as the most common change is from patterned genes integrating one or more AP factors to minimal overall expression.

**Table 2.** Patterning changes are strongly associated with increased TF binding.

	Low Expr to Patterned	Patterend to Uniform	Patterned to Low Expr	Uniform to Patterned
2.4×bcd	bcd,gt,kni,hkb,tll	—	—	bcd
0×bcd 0×hb 0 ×zld	gt,kni,tll bcd,gt,kni,tll —	bcd,cad,gt — —	— — bcd,cad,gt,kni,tll	bcd,kni bcd bcd,cad,gt,kni,tll

Using the genes with identified patterning changes in Figure 1, we performed a χ ^2^ test with a Bonferroni-corrected p-value of 0.05.

Furthermore, *bicoid* stands out as a major factor involved in AP patterning. In all of the mutant conditions except *bcd* and *zld* depletion, having a Bcd binding site is associated with an increase in patterned expression. In all of the conditions, a Bcd binding site is associated with a ubiquitous becoming patterned, and this pattern is often anterior expression.

In addition to patterning changes, some genes with ubiquitous localization actually showed the same response in absolute level as a result of both *bcd* depletion and over-expression. Of these genes, 1002 showed at least 1.5 fold higher expression on both conditions, and 414 showed a 1.5 fold decrease in expression. Such a scenario suggests that these genes are, at wild-type levels, tuned to a particular level of Bicoid expression.

It is difficult to reconcile increases of expression in the posterior with any local model of transcription factor action. Bcd protein is only present at approximately 5nM at 50% embryo length, and negligible levels more posterior ^29^. It is conceivable that Bcd activates a repressor gene somewhere in the anterior, which then diffuses more rapidly than Bcd to cover at least some of the posterior of the embryo. Nevertheless, there have previously been hints that Bicoid can function as far to the posterior as *hairy* stripe 7 ^30^.

### Genes are likely to change in similar ways in different mutant conditions

**Figure 8.**
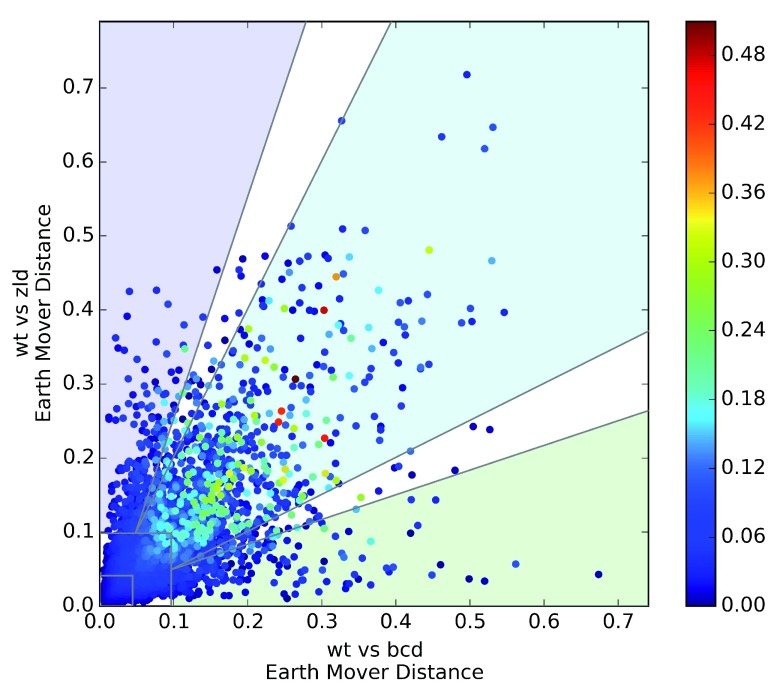
Genes that change in *bcd-* are likely to change in the same way in *zld-*, and *vice-versa.* Change versus the wild-type is plotted on the x and y axes. Each point is colored according to its ΔD score, calculated in Equation 1, in order to highlight genes that change differently between the two conditions.

We next asked whether patterning changes in one genotype could be used to predict whether the pattern changes in another. Therefore, we plotted the EMD between wild-type and the *bicoid* RNAi line on the X axis, and wild-type to the *zelda* GLC on the Y axis ( Figure 8). Unsurprisingly, the majority of genes did not change, but of those that did, only a small fraction of them changed in one condition but not the other (the blue and green regions near the axes). We grouped genes according to whether they were in the top 20% of the EMD distribution for each genotype independently, then performed a Pearson’s χ ^2^ test of independence of change in *bcd-* versus *zld-*. The result was highly significant (p<1×10 ^-100^), with the largest overrepresentation coming from the case where both changed. Repeating this across all combinations of wild-type and two other mutant genotypes yielded the same results: in every case, there were between 2.2 to 2.7 times as many genes that changed in both categories as would be expected ( Figure S3).

Of these genes that do change in both conditions, the majority changed in effectively identical ways. We computed a modified EMD that down-weights genes that are very similar to wild-type in at least one mutant genotype:

Δ *D* = EMD( *M*1, *M*2)—|EMD( *M*1, *WT*)—EMD( *M*2, *WT*)| (1)

where EMD(x, y) is the Earth Mover’s Distance between identically staged embryos of genotype x and genotype y. Even among only the set of genes that change in both conditions, ΔD is small (mean of 3.5%, 95th percentile of 11.9%)—equivalent to a shift of the entire pattern by about 1 or 2 slices in either direction. However, there are 13 genes that change differently between wild-type, *bcd-*, and *zld-* (ΔD>20%). These genes have noticeably different patterns in all three genotypes ( Supplemental Figure S4).

### Differential response to mutation is strongly associated with transcription factor binding

We sought to understand what is different about genes with a high ΔD, compared to those that change in response to wild-type, but have a low ΔD (that is, those that change in the same way in response to distinct mutant conditions). We found that genes with a high ΔD score were strongly enriched for a number of TF binding sites ( Table 3 and Table S1).

**Table 3.** TF binding is enriched near differentially changing genes between WT, *bcd*-, and *zld*-.

	odds ratio	base freq	p-value
bcd	4.69	17.40%	1.2e-09
kni	4.19	3.01%	0.000176
gt	3.79	19.00%	2.81e-08
cad	3.17	31.10%	6.73e-08
kr	3.1	56.58%	1.6e-07
tll	2.97	7.64%	0.000597
hb	2.21	39.53%	0.000143
hkb	2.1	23.86%	0.00115

χ ^2^ test results for TF binding within 10kb of the TSS for the wild-type/ *bcd*-/ *zld*- three-way comparison. We examined the top 50 genes by ΔD, compared to the 200 genes closest to the median ΔD of genes that change in response to both mutations. Base frequency indicates the fraction of genes expressed at this time point with at least one ChIP peak for that TF. In this comparison, only Dichaete and Zelda binding were not significant at a Bonferroni-corrected p-value of 0.05.

**Figure 9.**
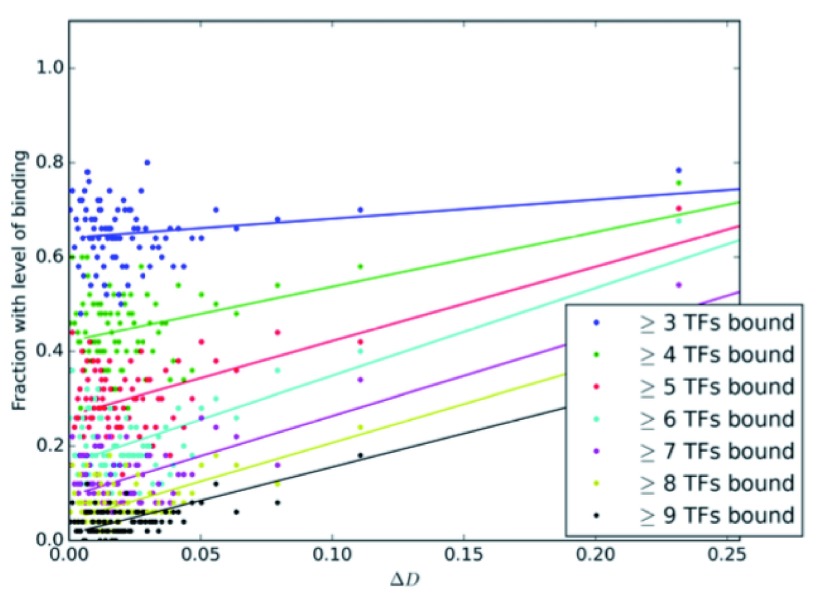
Higher ΔD scores are correlated with increased combinatorial binding. We grouped genes into non-overlapping windows of 50 genes by ΔD score, and calculated the fraction of those genes with at least 3,4, . . . *etc.* of the 10 early AP TFs bound (including Zld). We also plotted a simple linear regression on the binned points.

Next, we binned genes by ΔD score, then examined trends in combinatorial transcription factor binding. As ΔD score increases, genes are more likely to be bound by multiple TFs ( Figure 9). Due to the high background rate of binding, assaying the presence of at least 3 factors is not readily able to distinguish between genes with high and low ΔD’s, as nearly 70% of all genes expressed have at least 3 TFs bound. Assaying for the presence of more factors is better able to identify which genes are likely to change, and the top 50 genes all have at least 8 factors bound.

We sought to understand the extent to which genes with the same pattern of upstream regulators had the same responses to perturbation. We grouped genes according to the complement of ChIP-validated TF binding sites near that gene, then examined the patterning changes. Although with 10 different TFs there are potentially over one thousand distinct combinations of binding patterns, in practice the dense, combinatorial patterns found around patterning enhancers reduces this set to a much more manageable 157 different combinations, of which only 52 had at least 30 genes.

**Figure 10.**
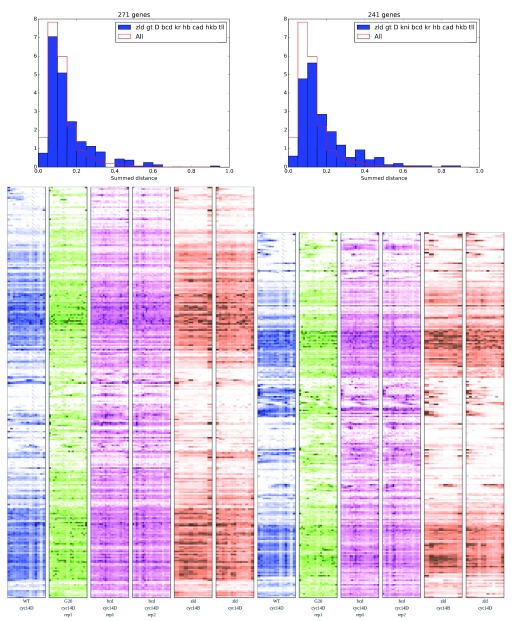
Identical binding patterns have a wide range of patterned responses.

Within these sets of genes with similar TF binding profiles, we then asked whether the distribution of patterning changes was any different from the distribution of patterning changes for all genes. For each gene, we summed the EMD scores for the 2.4× *bcd*, *bcd-*, and *zld-*, then performed a KS-test between the summed EMD scores of genes with a given binding pattern and the summed scores for all expressed genes. We found only 2 binding patterns with a Bonferroni-corrected p-value less than .05. Both of these sets were highly bound, and they were also very similar to each other in their binding, differing only in the presence of a Knirps (Kni) binding site ( Figure 10).

Despite the similar binding patterns near these genes, there is a wide range of responses. The wild-type expression patterns run nearly the complete gamut, including uniform expression, anterior stripes, posterior stripes, and central expression domains. Additionally, the presence of a Kni site seems to yield an increased number of genes with an anterior expression domain.

## Discussion

We have generated a dataset that is unparalleled in its coverage assaying patterning changes in mutant conditions. When these patterning mutants have been described previously, either major morphological readouts like cuticle staining or *in situ* hybridization has been used to illustrate the effects on downstream target genes ^12, 16, 31^. However, *in situ* hybridization suffers from a strong selection bias in the genes that are chosen. By assaying spatial differences in the patterning of every gene in the genome, we demonstrate the full effect that these TFs have on developmental gene expression networks.

Despite the importance of the factors we chose for establishing spatially and temporally correct patterning, only a relatively small number of genes have significant expression pattern changes. Many of the targets that do show clearly abnormal expression patterns are, themselves, key transcription factors. This suggests that, even though key, maternally provided patterning factors bind to thousands of places throughout the genome ^28^, many of those binding sites are not functional in any meaningful sense. Certainly some of this binding is due to artifacts in the ChIP data, and even reproducible, non-artifactual binding should not be confused with function ^32, 33^. However, the fact that genes near binding sites for multiple factors tend to have more complicated responses to mutation suggests that there is some truth to the idea that gene regulation in complex animals tends to be combinatorial, even if the ChIP data are imperfect.

We were surprised how much proper *bicoid* expression seems to be required for proper patterning at all points along the embryo, not just in the anterior. The fact that *bcd* is normally understood to be an activator, while the plurality of genes with higher, ubiquitous expression in the mutant are normally localized to the anterior in wildtype suggests that this is normally mediated through one or more repressors that depend on *bcd*. As one of three TFs overrepresented at genes with this phenotype, *gt* is likely to be involved in this global derepression, but since it is itself neither ubiquitous throughout the embryo nor universally bound at the genes that change, it is likely not the only player.

The mutants we examined seemed to produce very similar changes in their downstream targets, despite the wild-type TFs having widely varying spatial distributions. Our initial expectation was that there would be many more ways to fail to properly pattern expression, and that different mutations would have different average effects from each other. Indeed, relying on different mutations having different responses has been the key to genetic analysis of fine scale patterns such as the *eve* stripes ^17, 34– 36^. Although averaging across the most different genes in a mutant genotype does yield different patterns ( Figure 6), for any given gene excursions from the correct spatial expression pattern seems to be largely canalized ( Figure 8). This seemingly-canalized expression change may be a consequence of the types of genes we can easily measure patterning changes among—we cannot resolve individual pair-rule stripes, for instance—so genes with coarser patterns may be more likely to have a single “failure” phenotype, as compared to those with finer patterns, which have more layers of regulation to perturb.

We do recognize a number of distinct limitations of this dataset towards predicting gene expression change as a function of mutation. The spatial resolution is still much coarser than *in situ* hybridization based experiments. This is especially concerning near regions where there are fine stripes of expression, which cannot be resolved between adjacent slices, or at regions where there is a transition between expression domains, where it is possible that the slicing axis is not perfectly aligned with the domain border. Finally, it is worth remembering that especially in the later stages examined, the gap gene positions will also be perturbed, so any observed changes in pattern positioning is likely to be a combination of direct effects and downstream effects of the original mutation.

A number of recent studies have used various technical or experimental techniques to improve the resolution of RNA-seq maps of gene expression in developing embryos. Iterated sectioning of different embryos in all three dimensions can be deconvolved to yield estimates of the original pattern ^37^. Similarly, sequencing mRNA from dissociated nuclei allows for the maximum possible spatial resolution, assuming the original location of those nuclei can be estimated ^38, 39^. While these approaches are worthwhile for establishing a baseline map of expression patterns in wild-type embryos, the expense of sequencing still makes single-dimensional studies worthwhile. Furthermore, the single-cell approaches in Satija *et al.* ^38^ and Achim *et al.* ^39^ require some prior knowledge of spatial gene expression, which may be significantly perturbed in patterning mutants. Other approaches for multiplexed *in situ* profiling of mRNA abundance have been described, but are not yet cheap or reliable enough to be readily useful for screening mutants ^40, 41^.

Additionally, the time and expense required for a single individual necessarily means that we have profiled only a small number of individuals. We were therefore careful to choose only highly penetrant mutations for analysis, and to choose individuals at as similar staging as we could. However, even for genes with a consistent, precise time-dependent response between individuals, the differences in staging are likely to be a significant contributor to variation. Furthermore, we only examined two relatively distant time points in this study (approximately 45 minutes apart), making comparisons across time fraught at best.

Nevertheless, this experiment suggests a number of genes for more detailed follow up studies. As our predictive power for relatively well-studied model systems, such as the *eve* stripes improves, it will be especially important to take these insights to other expression patterns in the embryo. The risk of over-fitting increases with the depth of study of any particular model system, even if any given study is relatively well controlled. Therefore, by demonstrating that particular insights hard-won in these model systems are broadly applicable, we can gain some confidence in the results, and we approach having a rigorous, broadly applicable predictive model of gene regulation.

Ultimately, we believe more datasets addressing chromatin state in response to different conditions will be necessary for accurate prediction of spatial responses to mutation. In a ChIP-seq dataset on embryos with different, uniform levels of *bicoid* expression, hundreds of peaks seem to vary with differing affinities to Bcd protein (Colleen Hannon and Eric Wieschaus, personal communication, March 2015). The zygotically expressed genes near these differential peaks also have different spatial localization in wild-type, and different average responses to the mutants presented here. In addition to spatially resolved expression measurements, spatially resolved binding and chromatin accessibility data will likely be necessary. While ChIP-seq experiments currently require several orders of magnitude more input material than can be reasonably collected from spatially resolved samples, recent method developments in measuring chromatin accessibility have shown that it is possible to collect data from as few as 500 mammalian nuclei ^42^. A similar amount of DNA is present in a single *Drosophila* embryo, which suggests that spatially resolved chromatin accessibility data may be achievable.

## Materials and methods

### Fly lines, imaging, and slicing

*Zelda* germline clone flies (w zld- FRT/FM7a; His2Av RFP) were a gift of Melissa Harrison, and were mated and raised as described previously ^10^. Embryos were collected from mothers 3–10 days old.

The construction of the *bcd* and *hb* RNAi flies has been described previously ^43^ and were obtained from the DePace Lab at Harvard Medical School. Briefly, we generated F1s from the cross of maternal tubulin Gal4 mothers (line 2318) with UAS-shRNA- *bcd* or UAS-shRNA- *hb* fathers (lines GL00407 and GL01321 respectively), then collected embryos from the sibling-mated F1s. In order to take advantage of the slowed oogenesis and resulting greater RNAi efficiency, we aged the F1 mothers for approximately 30 days at 25°C.

The *bcd* overexpression lines were a generous gift of Thomas Gregor at Princeton University. We used line 20, which has 2.4× wild-type levels of eGFP-Bcd fusion. Flies were kept in uncrowded conditions, and embryos were collected at 25°C from 3–7 day old mothers.

We washed, dechorionated, and fixed the embryos according to our standard protocol (see 44), incubated in 3 µM DAPI for 5 minutes, washed twice with PBS, and then imaged on a Nikon 80i microscope with a Hamamatsu ORCA-Flash4.0 CCD camera. We did not DAPI stain the *zld-* embryos because they had a histone RFP marker. After selecting embryos with the appropriate stage according to density of nuclei in histone-RFP or DAPI staining and membrane invagination for the cycle 14 embryos, we washed embryos with methanol saturated with bromophenol blue (Fisher), aligned them in standard cryotome cups (Polysciences Inc), covered them with VWR Clear Frozen Section Compound (VWR,West Chester, PA), and froze them at -80C.

We sliced the embryos as in Combs and Eisen ^44^. Single slices were placed directly in non-stick RNase-free tubes (Life Technologies), and kept on dry ice until storage at -80C.

### RNA extraction, library preparation, and sequencing

We performed RNA extraction in TRIzol as previously ^44^. All RNA quality was confirmed using a BioAnalyzer 2100 RNA Pico chip (Agilent).

We generated libraries of the *zld-* embryos using the TruSeq mRNA unstranded kit (Illumina). As described previously, we added in 70 ng of yeast total RNA as a carrier and performed reactions in half-sized volumes to improve concentration ^44^.

We generated libraries from the RNAi and overexpression embryos using the SMARTseq2 protocol; we skipped the cell lysis steps because RNA had already been extracted ^45, 46^. As described previously, tagmentation steps were performed at 1/5th volume to reduce costs ^46^.

### Data analysis and deposition

All data was compared to FlyBase genome version r6.03 ( ftp://ftp.flybase.net/releases/FB2014_06/). Mapping was performed using RNA-STAR v2.3.0.1 ^47^, and expression estimates were generated using Cufflinks v2.2.1 on only the *D. melanogaster* reads ^48^. Reads from Combs and Eisen ^44^ were re-mapped to the new genome version. When carrier RNA was used (data from Combs and Eisen ^44^ and the *zld-* embryos), we discarded as ambiguous reads with 3 or fewer mismatches to prefer one species or the other. Due to the extensive divergence between the yeast carrier RNA and fly target RNA, the vast majority of mapped reads (>99.99%) were unambiguous as to the species of origin. After mapping, we removed samples that had fewer than 500,000 *D. melanogaster* reads and samples with less than a 70% mapping rate when no carrier RNA was used; no other filtering or corrections were performed.

Specific analysis code was custom-written in Python 2.7.6. Custom analysis and heatmap generation code is available from https://github.com/petercombs/EisenLab-Code. All analyses presented here and all data figures were made using commit 2c144be (doi: 10.5281/zenodo.160787). EMDs were calculated using the python-emd package by Andreas Jansson (no version number available, version used archived under doi: 10.5281/zenodo.160797). Violin plots, histograms, and scatter plots were made using Matplotlib v1.4.2 and Numpy v1.9.2, ^49– 51^. Linear regressions ( stats.linregress), Kolmogorov-Smirnov tests ( stats.ks_2samp), and *χ* ^2^ tests ( stats.chi2_contingency) were performed using Scipy v 0.14.0.

Newly generated sequencing reads have been deposited at the Gene Expression Omnibus under accession GSE71137. Additional files and a searchable database are available at http://eisenlab.org/supplements/combs2016/.

## Data availability

The data referenced by this article are under copyright with the following copyright statement: Copyright: © 2017 Combs PA and Eisen MB

Newly generated sequencing reads have been deposited at the Gene Expression Omnibus under accession GSE71137 ( https://www.ncbi.nlm.nih.gov/geo/ query/acc.cgi?acc=GSE71137).

Custom analysis code: https://github.com/petercombs/EisenLab-Code

Archived custom analysis code at the time of publication: http://dx.doi.org/10.5281/zenodo.160787

## Supplementary material

**Supplemental Figure 1: Adjacent slices are more similar than distant ones.** Violin plots of the Spearman Rank correlations between adjacent slices and pairs of slices separated by more than one third of the embryo length.

**Supplemental Figure 2: Figure 7 normalized to expression in wild-type cycle 14D highlights absolute expression level changes.** Slices with higher expression are clipped to the maximum expression in wild-type.

**Supplemental Figure 3: Genes that change tend not to change in only one condition.** Three-way comparisons, as in Figure 8, between wildtype and the remaining combinations of *bcd* depletion, *bcd* overexpression, *hp* depletion, and *zld* depletion. The wildtype-mutant comparison is indicated on each axis, and the color indicates the ΔD score between the two mutants. G20 indicates the bicoid overexpression line, line #20 from 52.

**Supplemental Figure 4: Only a handful of genes change differently between the different conditions.** In the WT vs *bcd-* vs *zld-* three- way comparison, only 13 genes had a ΔD score above 20%. Thumbnails indicate wild-type pattern in blue, *bcd-* pattern in both replicates in pink, and *zld-* pattern in orange. All expression is scaled to the highest in each individual.

**Supplemental Table 1: TF binding is enriched near differentially changing genes across all three-way comparisons.** χ ^2^ test results for TF binding within 10kb of the TSS for the indicated three-way comparison. We examined the top 50 genes by ΔD, compared to the 200 genes closest to the median ΔD of genes that change in response to both mutations. Base frequency indicates the fraction of genes with at least one ChIP peak for that TF and that are expressed at this time point in all three conditions.
