# Supplementary figures and images for: Genome-wide measurement of spatial expression in patterning mutants of Drosophila melanogaster
Source: F1000Res. 2017 Jan 12;6:41. [Version 1] doi: 10.12688/f1000research.9720.1 (PMC5325077; doi:10.12688/f1000research.9720.1)

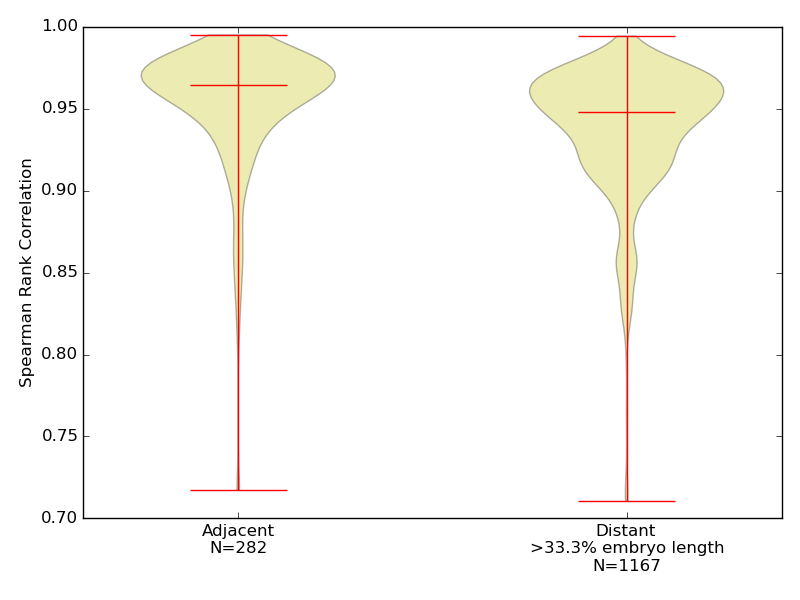

Supplement: Supplementary file 1 [file f1000research-6-10478-s0000.tgz › e6106648-c5aa-44e2-bfb2-98e3e6420058.png]

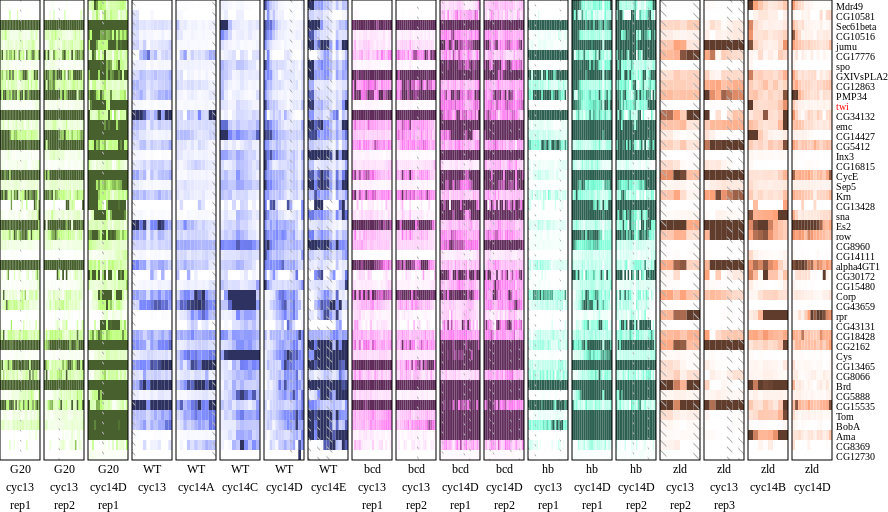

Supplement: Supplementary file 2 [file f1000research-6-10478-s0001.tgz › b6b069f9-b160-468c-840e-3befd2ad2a93.png]

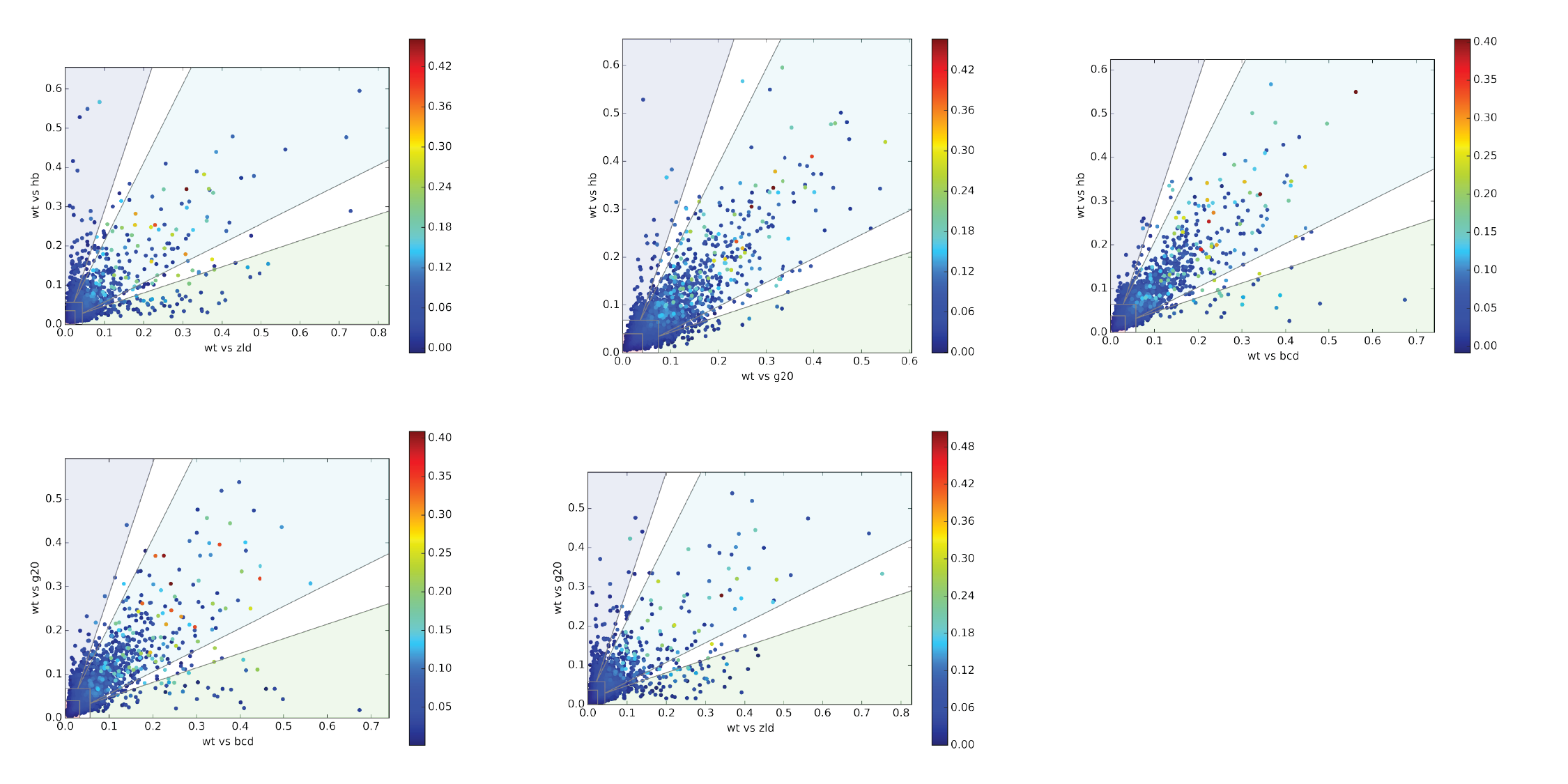

Supplement: Supplementary file 3 [file f1000research-6-10478-s0002.tgz › 478b28b9-bc37-4116-b58c-2eba30694a9e.png]

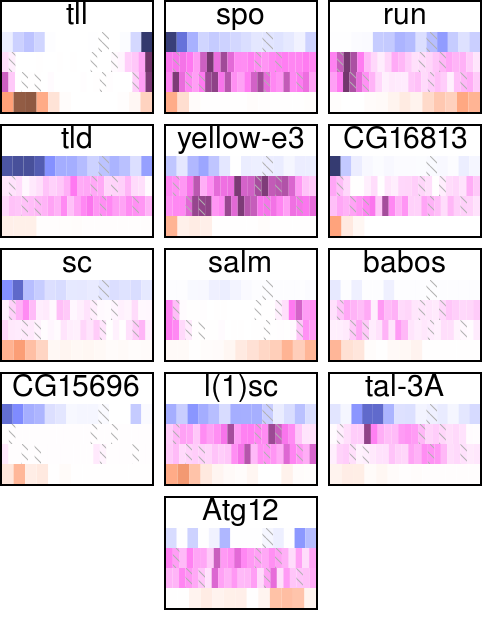

Supplement: Supplementary file 4 [file f1000research-6-10478-s0003.tgz › dfd182a9-e9f3-465e-bbb0-8a2c36bd579a.png]
