# Supplementary material for: Genome-wide measurement of spatial expression in patterning mutants of Drosophila melanogaster
Source: F1000Res. 2017 Jan 12;6:41. [Version 1] doi: 10.12688/f1000research.9720.1 (PMC5325077; doi:10.12688/f1000research.9720.1)
Supplement: Supplementary file 5 [file f1000research-6-10478-s0004.tgz › d30dd912-c6db-43c1-8596-280d010aec9e.pdf]

Table S1. TF binding is enriched near differentially changing genes across all three-way comparisons.

	odds ratio	base freq	p-value
WT vs *bcd-* vs 2.4× *bcd*			
kni	16.4	3.28%	8.93e-07
tll	5.5	8.08%	7.72e-05
bcd	4.1	17.58%	9.24e-05
gt	3.47	19.55%	0.00053
hkb	3.1	24.15%	0.0016
cad	3.09	31.98%	0.00067
WT vs *bcd-* vs *hb-*			
kni	11.0	3.34%	0.00297
tll	5.31	8.15%	0.000671
bcd	3.29	17.67%	0.00183
D	0.281	90.70%	0.00251
WT vs *zld-* vs 2.4× *bcd*			
kni	12.3	3.05%	3.63e-07
tll	11.3	7.67%	3.91e-11
cad	7.05	31.20%	1.62e-08
bcd	6.74	17.55%	7.02e-09
gt	4.56	18.99%	5.95e-06
kr	4.56	56.67%	8.79e-05
hb	3.95	39.74%	4.62e-05
WT vs *zld-* vs *hb-*			
kni	9.33	3.04%	0.000162
bcd	7.82	17.51%	6.34e-10
gt	6.14	19.01%	7.57e-08
tll	5.05	7.67%	0.000156
cad	4.77	31.01%	2.05e-06
kr	3.41	56.61%	0.00102
hb	2.84	39.68%	0.00186
WT vs 2.4× *bcd* vs *hb-*			
tll	18.5	8.16%	1.19e-07
kni	8.95	3.32%	0.00169
gt	4.27	19.57%	3.32e-05
bcd	3.78	17.76%	0.000173
hb	2.9	40.61%	0.00147
cad	2.87	31.72%	0.00152

χ^2 test results for TF binding within 10kb of the TSS for the indicated three-way comparison. We examined the top 50 genes by Δ D, compared to the 200 genes closest to the median Δ D of genes that change in response to both mutations. Base frequency indicates the fraction of genes with at least one ChIP peak for that TF and that are expressed at this time point in all three conditions.
