# Supplementary material for: Genome-wide measurement of spatial expression in patterning mutants of Drosophila melanogaster
Source: F1000Res. 2017 Jan 12;6:41. [Version 1] doi: 10.12688/f1000research.9720.1 (PMC5325077; doi:10.12688/f1000research.9720.1)
Supplement: Peer review history

# Peer review: Referee response for version 1

**DOI:** 10.5256/f1000research.10478.r19328

**Published:** 2017-02-22

**Authors:** Angela H. DePace

**Affiliations:** 1 Department of Systems Biology, Harvard University, Boston, MA, USA

This paper by Combs and Eisen examines spatially patterned gene expression in Drosophila embryos in response to mutation of key developmental transcription factors. The unique contribution of this work is the scale of their measurement — mRNA expression assessed genome-wide in slices along the anterior/posterior axis of individual embryos. Typically, genome-wide measurements are not spatially resolved; conversely spatially resolved measurements are typically only made for a handful of genes at once. This technique was described and validated in a previous paper. Here they apply it to characterize the response to mutations of bicoid, zelda and hunchback.

 The dataset is valuable and the underlying data collection is well described. There results also point to a number of interesting conclusions. In particular, it’s notable that despite being bound widely across the genome, knocking down these TFs produces only modestly affects patterning on average. It is also notable that Bicoid knockdown can affect patterning in the posterior, where it is not expressed. My suggestions for improving the paper are below.

1. The introduction does not introduce a biological question that they will be able to address with their method, but instead focuses on the novelty of the measurement. They state that “Given the crucial roles of each of these factors in spatial patterning, we expected that perturbing their levels would lead to widespread direct and indirect effects on patterned genes.”  It would be useful to expand on this hypothesis in terms specific to the data they will collect and how they will analyze it. Which features of the data would confirm or refute this hypothesis? What will they find interesting or surprising? For example, they do some of this in the discussion. “Our initial expectation was that there would be many more ways to fail to properly pattern expression, and that different mutations would have different average effects from each other.” Can their expectation be made more specific given the diversity of patterns they can detect in WT embryos? Why did they have this expectation about the average effects when so many genes are unpatterned? Bringing some of the more interesting points in the discussion to the introduction to frame the paper would motivate the reader to understand the various analyses.
2. The quality, reproducibility and structure of the data should be more explicitly discussed, and used to explain their choice of analytical strategy. For example, they justify their use of the Earth Mover’s Distance to examine expression patterns: “Because there are a different number of slices both between the wild type and mutant flies, and between replicates of the mutant flies…”  Does this sentence mean that there are more slices per embryo for a WT versus a mutant embryo, or that there are in aggregate more mutant slices than WT slices (because they measured more mutants)? How does this impact their choice of analyses? Given the structure of the data and its variability, what are the limits of analysing effects on single genes (which would be more familiar to developmental biologists)? Simple language could help more readers appreciate the utility and limits of their data for future analyses.
  In addition, a narrative description of the EMD would be helpful. The authors state : “...we decided to use Earth Mover’s Distance (EMD) to compare patterns[20]. This metric captures intuitive notions about what kinds of patterns are dissimilar, yielding higher distances for dissimilar distributions of RNA, and zero for identical distributions.”  This states that the range will go from 0 (identical) to “higher” (dissimilar) but does not justify their use of this metric. It would be useful to explain the value of this choice of metric over available alternatives.
3. The figure legends and titles could be more informative. In general, the legends state only what is depicted and does not point the reader to the most relevant comparisons. While this isn’t strictly necessary, it can help readers who are unfamiliar with the particular type of plot used. Figure 6 refers to panels A - D, which are not labeled in the figure. The legend of Figure 7 is a single sentence stating “Each embryo is normalized separately.” In some cases, the grammar of the titles is difficult to parse. For example, the title of Figure 4 is: “Distributions of patterning differences show that mutants have wide-spread subtle patterning effects and more genes with large patterning differences than replicates.” The clause of this sentence is unclear. The title of Figure 5 is similarly difficult to parse.

I have read this submission. I believe that I have an appropriate level of expertise to confirm that it is of an acceptable scientific standard.

# Peer review: Referee response for version 1

**DOI:** 10.5256/f1000research.10478.r19327

**Published:** 2017-02-20

**Authors:** Thomas Gregor

**Affiliations:** 1 Department of Physics, Princeton University, Princeton, NJ, USA

The paper by Combs and Eisen extends the previously published dataset of RNA-seq measurements in cryo-sliced Drosophila embryos to mutants of patterning genes. As the authors state, most common methods for gene expression measurements are either limited in throughput or average over spatial coordinates. The development and application of an experiential approach addressing this gap is therefore of high interest. Datasets produced with this approach are an extremely valuable resource to diverse studies in the community.   In the presented application the author go beyond the characterization of wild type embryos to the examination of Zelda, Hunchback and Bicoid mutants. This study thus offers the opportunity to examine the contribution of these key factors to the formation of spatial patterns of gene expression in the early embryo.  That said, the description of the analyses carried out and the presentation of the results could be improved to further facilitate the usefulness of this dataset and of the approach.

 While the analyses focuses on comparison between samples (and here a single slice from a single embryo at a single time point can be considered a sample) the authors provide little information on how these comparisons are done. It would be beneficial to specifically note what type of assumptions are made in the analysis, how samples are normalized with respect to one another and how these normalizations influence the quantitative nature of the statements that can be made. For example, does the analysis performed implicitly assume that the total number of RNA produced (and captured) in each slice is the same? Can the authors address more extensively how they deal with differences in the exact number and size of slices. As patterns are then normalized to their maximal level prior to comparison between conditions, it seems genes are not assumed to maintain the same maximal activity yet claims about absolute changes in level cannot be made; can the authors clarify if this is indeed the case. Given these issues, it would be useful to include a more explicit description how a “uniform” vs a “patterned” gene is defined. Maybe the authors could show this graphically using an average plot of all of the heat map data for the genes classified this way. A clarification of the quantitative meaning of phrases as a “greatly perturbed pattern”, “Higher expression everywhere” or “same response in absolute level”, would help elucidate the insights obtained from the data.

 It would also be useful if the authors provided a more extensive explanation of their usage of EMD, a method that was not employed in their analysis in their 2013 paper. This can be included the in methods/supplemental information. How would the similarity in the changes between genes/mutants ascertained with this measure compare to others measures (e.g. correlating patterns or changes in patterns). In Figure 10, could the summation of the EMD scores mask some of the signal? (is the result the same for each EMD score?). What is the initial diversity in patterns among genes that belong to the same set in this analysis (i.e. sharing a similar ChIP binding pattern)? The authors present figure 10 as an investigation of how genes with binding sites for the same TFs respond to perturbation (assuming “patterned responses” means responses to mutation), but only discuss their wild-type patterns. This is a good example of the heatmaps having too much information to be illustrative. It is difficult to relate the EMD to physical changes in patterns of target genes. It's not obvious, based on the barplot and the heatmaps in the figure, what it is about these expression patterns that resulted in these two groups of genes being significantly different from each other. Maybe a better explanation of the EMD would clarify this. It is not directly clear mathematically how EMD measures how quantitatively different two groups are. For example, only two cohorts of TFs showed a significant difference in the EMD of the genes in their group; the histograms show the distributions of EMDs in genes with each cohort of TF binding sites vs. the EMDs in the total dataset; it looks like the two significantly different groups are both skewed toward higher EMDs than the total dataset, but it is unclear what makes them different from *each other,* based on the figure and the explanations provided.

 Additionally, despite showing the replicates in most figures, I still find it hard to assess to what extent are patterns variable between replicates (due to both biological and technical reasons) and what is the magnitude of these differences compared to those observed in the mutants. For instance in Figure 4A, do the zld replicates show bigger differences than most of the other presented comparisons? How is the significance of the differences between the presented distributions should be assessed?

 While the large heatmaps are useful for initially appreciating the scope of the method and for showing overall patterns, it is difficult to assess the change in a pattern for a specific gene (or subset of genes). Possibly presetting the data in each mutant genotype as the change from wild-type would help in that regard?

 The legend of the figures can be more elaborate and clearer. For example I am not sure I understand the analyses presented in Figure 6. Is the pattern or the change in pattern averaged and presented? The replicates in this figure seem quite different from one another, and it is hard to see how the “changes recapitulate known TF localization and function”, as stated in the title. Isn’t averaging over the 100 genes with largest changes masking the patterning changes (unless they are highly similar among all of these genes)? Only some of the colors presented are mentioned in the legend. There is a reference to panels A-D but these do not appear in the figure. Some figures include “G20”, yet only in a supplementary figure legend it is specified that it indicates the bicoid overexpression line.

 Finally, as the method is still rather new, and it is very hard to assess experimental error, can the authors pick one of the more surprising observations, like a gene with increased expression in the posterior in bcd mutant, and provide a control experiment with an alternative method (e.g. *in situ*) demonstrating at least a qualitative agreement?

I have read this submission. I believe that I have an appropriate level of expertise to confirm that it is of an acceptable scientific standard.
